# Dose-Dependent Effects of Turmeric (*Curcuma aromatica* S.) Starch on Colonic Fermentation in Rats

**DOI:** 10.3390/metabo14110572

**Published:** 2024-10-24

**Authors:** Ekanayake Mudiyanselage Asanka Chamara Ekanayake, Ryota Ishii, Ryuji Nagata, Ken-ichiro Shimada, Kyu-Ho Han, Michihiro Fukushima

**Affiliations:** Department of Life and Food Sciences, Obihiro University of Agriculture and Veterinary Medicine, Obihiro 080-8555, Hokkaido, Japan; s21180005@st.obihiro.ac.jp (E.M.A.C.E.); s18120011@st.obihiro.ac.jp (R.I.); rnagata@obihiro.ac.jp (R.N.); kshimada@obihiro.ac.jp (K.-i.S.); fukushim@obihiro.ac.jp (M.F.)

**Keywords:** acetate, gut microbiota, turmeric starch, visceral fat

## Abstract

Background; Turmeric starch (TS) has gained significant attention due to its potential health benefits. Rich in resistant starch (RS) and higher in phosphorus, TS is anticipated to possess properties of high-phosphorus-type RS. Objectives; To understand the host physiology of TS, this study investigated the dose-dependent effects of TS on colonic fermentation in rats. Methods; Four experimental diets containing different levels of TS (5%, 10%, and 20% *w*/*w*) were formulated and fed to male Fischer 344 rats for two weeks and compared with rats fed a 0% TS diet (TS0). Results; Results showed that increasing the dose of TS resulted in reduced body weight gain, lower visceral tissue weight, and increased cecal mucin and IgA levels compared with the TS0 group. Further, fecal dry weight increased dose-dependently parallel to the starch excretion rate. Higher doses of TS resulted in increased short chain fatty acid (SCFA) production, specifically cecal acetate content, as well as in a dose-dependent decrease in the cecal pH level. However, this study did not observe a positive effect of TS on colonic alkaline phosphatase (ALP) activity, and the impact on small intestinal ALP activity remains unclear. Notably, beneficial bacteria such as the family *Oscillospiraceae*, genus *Lachnospiraceae NK4A136* group, and *Ruminococcus* spp. were found to have been enriched in the TS-fed groups, further supporting the beneficial effects of TS on gut microbiota and SCFA production. Additionally, the genus *Mucispirillum*, which is known to possess beneficial and opportunistic pathogenic traits under immunocompromised states, was found in the TS-fed groups. Conclusions; According to these results, it is clear that TS served as a prebiotic substrate in rats, with a notable modulation of the microbial composition.

## 1. Introduction

Turmeric (*Curcuma aromatica* S.), a well-known spice, has been extensively studied for its potential health benefits. The primary active compound in turmeric is curcumin, which has anti-inflammatory, antioxidant, and anticancer properties [1]. However, in addition to curcumin, turmeric contains starch. While turmeric starch (TS) has received less attention compared with curcumin, emerging evidence suggests it may also have beneficial effects on health, particularly in the context of colonic fermentation [2,3], and it has been reported that TS is a B-type starch which possesses higher resistant starch (RS) content [4]. Specifically, TS is known for its substantial phosphorus content (~5000 ppm), similar to potato starch [5]. Consequently, research has indicated a negative relationship between phosphorus content and starch digestibility when subjected to enzyme-mediated hydrolysis after gelatinization [6].

Dietary fiber-rich diets modulate energy metabolism through the breakdown by gut bacteria, leading to the production of short chain fatty acids (SCFAs) [7] and increasing the colonic alkaline phosphatase (ALP) activity [8]. SCFAs influence satiety, energy expenditure, adipogenesis, and glucose metabolism, all of which play a role in body weight regulation [9]. Previous studies have shown that the enzyme-resistant fraction derived from spent turmeric exhibits similar fermentation potential to other types of resistant starch, leading to significant increases in SCFAs in vitro [10]. Additionally, the consumption of whole turmeric powder has been associated with a decrease in body weight gain, reduced energy intake, and a reduction in the accumulation of visceral fat in rats. Moreover, it has been reported to lower serum triglyceride and lower hepatic total lipid levels in rats [2]. In addition, a study discovered that hydrothermally treated depigmented turmeric powder effectively boosts cecal fermentation in rats. This enhancement is attributed to the combined effects of fiber and RS, resulting in increased concentrations of acetate and butyrate [5]. The inclusion of spent turmeric powder at a concentration of 10% has been shown to inhibit intestinal lipid absorption and reduce overall energy intake [3].

These findings suggest the potential of turmeric and its byproducts to positively impact fermentation processes, lipid metabolism, and energy metabolism, highlighting their possible role in promoting health and preventing metabolic disorders. Understanding the dose-dependent effects of TS on colonic fermentation in animal models can provide valuable insights into its potential as a functional ingredient. The objective of this study is to investigate the dose-dependent effects of TS on colonic fermentation and the physiological effects in rats.

## 2. Materials and Methods

### 2.1. Turmeric Starch and Experimental Diet

Turmeric was supplied by Okinawaukondo Co., Ltd. (Okinawa, Japan). Starch from dry turmeric slices was recovered using a method that has been applied to several starchy crops, with modification [11]. Dried turmeric slices were ground into fine particles using a laboratory scale grinder (FM-1, Osaka Chemical Co., Ltd., Osaka, Japan) and suspended in water. The suspension was mixed in a laboratory scale blender (JMM-1020, Yamazen Corporation, Osaka, Japan) for 60 s, followed by filtration using a nylon mesh cloth. The filtered starch milk was wet sieved using a set of standard sieves (350, 75, and 50 µm). The sieve-passing fraction was allowed to sediment for 2 h; the upper layer of water was removed; the wet starch was washed with ethanol (70%, *v*/*v*) to remove the pigments; and the suspension was filtered through a glass filter (26G2, Asahi Glass Co., Ltd. Tokyo, Japan) with the aid of vacuum filtration. This filtration process was repeated multiple times until the color was completely removed from the starch. Finally, the isolated starch was dried for 24 h under ambient conditions.

The proximate composition of TS (Table 1) was determined according to the AOAC methods as follows: moisture (AOAC 925.10) [12], crude protein (AOAC 920.87) [13], crude lipids (AOAC 2002.02) [14], and ash (AOAC 923.03) [15]. RS content was quantified using the Megazyme Resistant Starch Assay Kit (Megazyme, Wicklow, Ireland) according to the manufacturer’s instructions. Briefly, the process involves using 0.1 g of starch samples, enzymatically removing digestible starch, isolating the resistant starch fraction, hydrolyzing it to glucose, and then quantifying the glucose content through a colorimetric reaction by measuring absorbance at 510 nm using a spectrophotometer (UV-1600, Shimadzu, Kyoto, Japan). The energy contents in the TS and the experimental diets were calculated as previously described [16]. Phosphate content in the TS was determined using an inductively coupled plasma atomic emission spectrophotometer (ICPS-8100, Shimadzu Co., Ltd., Kyoto, Japan). Samples for ICPS-8100 were prepared as previously described [11]. All chemicals used were of analytical grade. Four experimental diets, 0% *w*/*w* TS (TS0), 5% *w*/*w* TS (TS5), 10% *w*/*w* TS (TS10), and 20% *w*/*w* TS (TS20) were formulated based on the AIN-93G diet guidelines by Oriental Yeast Co., Ltd. (Tokyo, Japan) (Table 2).

### 2.2. Experimental Design

The animal experiment was conducted according to the guidelines of “Guide for the Care and Use of Laboratory Animals”, and all the procedures were approved by the Animal Care and Experiment Committee of Obihiro University of Agriculture and Veterinary Medicine (License No.:22-159). The rat strain, Fischer 344, was chosen as the experimental animal model in this study, as it has been previously used in nutrition and dietary research [17]. A total of 28 male Fischer 344 rats (7 weeks old) were purchased from Charles River Laboratories Japan Inc. (Yokohama, Japan). Each rat was individually housed in plastic cages under controlled environmental conditions (temperature: 23 ± 1 °C; relative humidity: 60 ± 5%; 12 h light/dark cycle). Prior to the start of the experiment, the rats were acclimatized for seven days on a standard rodent diet (CE-2, CLEA Japan, Inc., Tokyo, Japan). After acclimatization, the rats were randomly assigned to four similar body weight groups (180 ± 1 g) and fed one of the four experimental diets (≃25 g) with free access to ad libitum water (≃150 mL).

Weekly body weight gain and daily feed intake were measured. During the last two days of the experimental period, fecal pellets were collected and stored at −30 °C for the analysis of fecal starch content. After the experimental period of two weeks, the final body weight was measured and the rats were anesthetized by inhalation of isoflurane and euthanized by cervical dislocation assuring the minimal level of suffering. The cecum, liver, epididymal, and perirenal adipose tissues were excised and weighed. A portion of the cecal content (≃1 g) was diluted (×5) in distilled water for pH measurement [17], while the rest was stored at −80 °C.

### 2.3. Cecal Organic Acid Analysis

The cecal organic acid content in the rats was determined using HPLC (LC-10AD, Shimadzu, Kyoto, Japan). The supernatant obtained by centrifugation of the cecum content suspension at 12,000× *g* for 15 min at 4 °C was ultrafiltered using a TORAST-H ultrafilter (Shimadzu GLC, Kyoto, Japan) by centrifugation at 12,000× *g* for 10 min at 4 °C, followed by further filtration using a cellulose acetate membrane filter (0.45 µm, Toyo Roshi Kaisha Ltd., Tokyo, Japan) and used in HPLC. Analytical specifications were as follows according to [18]; column, RSpak KC-811 (8.0 mm × 300 mm, Shodex, Tokyo, Japan); eluent and flow rate, 2 mM HClO_4_ at 1 mL/min; column temperature, 47 °C; reaction reagent and flow rate, ST3-R (×10 diluted, Cat. No. F56120000, Shodex) at 0.5 mL/min; UV detector wavelength, 450 nm.

### 2.4. Analysis of Cecal Mucin Content

The cecal mucin fraction was isolated from diluted cecal suspension samples (≃1 mL) using the method previously described [19] and mucin levels were determined by the fluorometric assay using a fluorescence spectrophotometer (FP-6200, Jasco International Co., Ltd., Tokyo, Japan) at an excitation wavelength of 336 nm and measurement wavelength of 383 nm as previously described [20].

### 2.5. Analysis of Cecal Immunoglobulin A (IgA) Content

The IgA levels in the rat cecal contents were determined using a rat IgA ELISA quantitation kit (Bethyl Laboratories, Montgomery, TX, USA) according to the manufacturer’s instructions. Briefly, diluted cecal samples were thoroughly mixed using a vortex and centrifuged (12,000× *g*, 4 °C, 15 min) to obtain the supernatant for IgA analysis. IgA levels were quantified through a colorimetric reaction by measuring absorbance at 450 nm using a microplate reader (MultiskanTM FC, Thermo Fisher Scientific, Tokyo, Japan).

### 2.6. Analysis of Cecal Ammonia–Nitrogen Content

The cecal ammonia–nitrogen contents were determined using a commercially available kit (FUJIFILM Wako Pure Chemical Corporation, Osaka, Japan) according to the manufacturer’s instructions. Briefly, an aliquot from the cecal homogenate (0.1 M phosphate buffer, pH 5.5) was used for the analysis, and absorbance was measured at 630 nm using a spectrophotometer (UV-1600, Shimadzu, Kyoto, Japan).

### 2.7. Fecal Starch Content

The starch content in the previously mentioned feces (Section 2.2) was measured using the Megazyme Resistant Starch Assay Kit (Megazyme, Wicklow, Ireland) according to the manufacturer’s instructions. The starch excretion rate was calculated by the following equations:

Starch excretion rate = (*FS*/*SI*) × 100, where *FS* is the amount of fecal starch content over the last 2 days and *SI* is the amount of starch intake over the last 2 days of the experimental period.

### 2.8. Cecal Bacterial DNA Extraction, Next-Generation Sequencing, and Analysis of Microbiota

Cecal bacterial DNA was extracted from the non-diluted cecal content samples stored at −80 °C using the repeated bead beating plus column method as previously described [18]. The extracted DNA was then purified through sequential digestion with RNase and proteinase K using the QIAamp Fast DNA stool mini kit (Qiagen, Valencia, CA, USA) following the manufacturer’s instructions. The concentration and the purity of the extracted DNA were measured using a NanoDrop 2000c Spectrophotometer (Thermo Fisher Scientific, Tokyo, Japan). Finally, the concentration of the extracted and purified DNA was adjusted to 5 ng/µL using Tris-EDTA buffer (pH~8.0). Paired-end sequencing of 16S rRNA gene amplicons was performed using the Illumina MiSeq system (Illumina, San Diego, CA, USA) as previously described [18]. The retrieved raw 16S rRNA gene sequences (Supplementary; s1) were analyzed using the Quantitative Insight Into Microbial Ecology (QIIME2) version 2023.2 [21]. The generated biome table was normalized using an equal subsampling size of 33,960 sequences. The weighted UniFrac distance metric was employed for determining the β-diversity, and a principal coordinate analysis (PCoA) plot was generated using R Studio (version 2023.3.1). A linear discriminant effect size analysis (LEfSe) was performed at the genus taxonomical levels to identify the genera representing different groups using MicrobiomeAnalyst 2.0 [22]. The threshold of the logarithmic linear discriminant analysis (LDA) score for discriminative features was 2.0.

### 2.9. Analysis of Alkaline Phosphatase Activity

The intestinal mucosa samples were homogenized with 10 mM Tris-buffered saline (pH 7.3) containing 1% Triton X-100 (Sigma-Aldrich, Tokyo, Japan). The homogenate was centrifuged at 7000× *g* at 4 °C for 15 min [23]. The supernatant was used as an enzyme-enriched extract. ALP activity was measured using a Lab Assay ALP kit (BioAssay Systems, Hayward, CA, USA) and protein concentration was determined using a Bio-Rad protein Assay kit (Bio-Rad Laboratories, Hercules, CA, USA), according to the manufacturer’s instructions. Briefly, the dye reagent was added to the homogenized mucosa samples and standards, followed by a 15 min incubation at room temperature to allow color development. The absorbance was measured at 765 nm using a microplate reader (MultiskanTM FC, Thermo Fisher Scientific, Tokyo, Japan). Protein concentration in unknown samples was determined by comparing their absorbance to a standard curve generated from known protein concentrations.

### 2.10. Statistical Analysis

All zoometric and biochemical data are presented as mean ± SE. The statistical analysis was performed by one-way ANOVA followed by Dunnett’s post hoc test to compare all diets with the control group. Tukey’s post hoc test was performed to determine significant differences between the TS-fed groups. Correlations between the parameters were assessed using Pearson’s correlation analysis. Statistical differences in alpha diversity indices at feature levels, relative abundance of phyla, and genera among the four diet groups were analyzed using the non-parametric Kruskal–Wallis H test followed by the Bonferroni correction. The association between microbial abundance and biochemical data was tested using Spearman correlations analysis. Alpha diversity indices and relative abundances of genera are presented in box and whisker plots. The boxes show medians/quartiles, and whiskers extend to the most extreme value within 1.5 interquartile ranges. A *p*-value less than 0.05 was considered statistically significant. All statistical analyses were performed using SPSS software (version 29.0.0.0 (241)).

## 3. Results

### 3.1. Zoometric Parameters

The zoometric parameters are provided in Table 3. Upon completion of the experimental period, feed intake, body weight gain, and final body weight were not significantly different in the TS-fed groups compared with the TS0 group. However, the TS-fed groups showed significantly lower (*p* < 0.001) perirenal and epididymal adipose tissue weights in a dose-dependent manner compared with the TS0 group, while the TS20 group possessed the lowest perirenal and epididymal adipose tissue weights (Table 3). The fecal dry matter content and starch excretion were significantly higher (*p* < 0.01) in the TS-fed groups compared with the TS0 group, with the TS20 group exhibiting the highest fecal dry matter content and highest starch excretion rate. When comparing among the TS-fed groups, both fecal dry matter content and starch excretion were significantly increased (*p* < 0.05) in a dose-dependent manner, with the highest levels observed in the TS20 group.

### 3.2. Cecal Parameters in Rats

Cecal acetate content was significantly increased in a dose-dependent manner in the TS-fed groups compared with the TS0 group (*p* < 0.05). The propionate and butyrate contents did not show significant differences among the diet groups. Furthermore, cecal lactate content was significantly higher (*p* < 0.05) in the TS10 and TS20 groups compared with the TS0 group. Conversely, cecal succinate content was significantly lower in the TS10 and TS20 groups when compared with the TS0 group (Table 4). The cecal pH was significantly decreased (*p* < 0.05) dose-dependently compared with the TS0 group. Cecal tissue and cecal digesta weights were significantly increased dose-dependently (*p* < 0.05), indicating an increase in the cecal size in the TS-fed groups compared with the TS0 group. Further, when comparing between the TS-fed groups, the cecal pH and cecal digesta were significantly higher (*p* < 0.05) in the TS20 group compared with the TS5 group. The TS10 group did not show significant differences in these parameters compared with the TS5 and TS20 groups (Table 4).

### 3.3. Cecal Mucin, IgA, and Ammonia–Nitrogen

**Figure 1:**
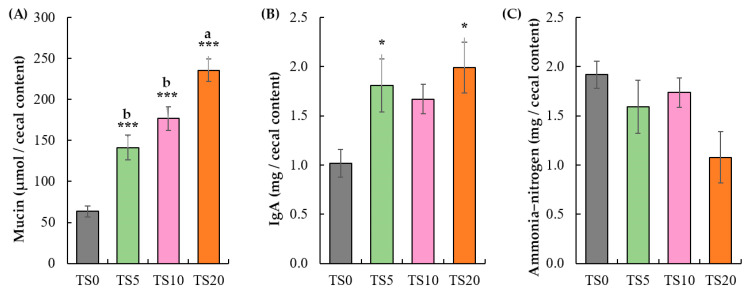
Cecal content: (**A**) mucin, (**B**) IgA, and (**C**) ammonia–nitrogen. Data are expressed as mean ± SE (TS0, 0% turmeric starch; TS5, 5% turmeric starch; TS10, 10% turmeric starch; TS20, 20% turmeric starch, *n* = 7). The statistical analysis was performed by one-way ANOVA, followed by Dunnett’s post hoc test (* *p* < 0.05, *** *p* < 0.001) to compare all TS diets with TS0 and by Tukey’s test (^a,b^ *p* < 0.05) to compare between TS-fed groups.

Cecal mucin content (Figure 1) was significantly increased (*p* < 0.001) in a dose-dependent manner in the TS-fed groups compared with the TS0 group. Among the TS-fed groups, the TS20 group showed a significantly higher (*p* < 0.05) cecal mucin content while TS5 and TS10 did not show a significant difference between them. The cecal IgA level (Figure 1) was significantly higher (*p* < 0.05) in the TS5 and TS20 groups compared with the TS0 group. Additionally, the IgA level in the TS10 group was comparatively higher (*p* = 0.10) than that in the TS0 group. Cecal ammonia–nitrogen content (Figure 1) did not show a significant difference among the diet groups. However, the TS20 group showed a comparatively low (*p* = 0.08) cecal ammonia–nitrogen content.

### 3.4. Microbial Diversity and Abundance

**Figure 2:**
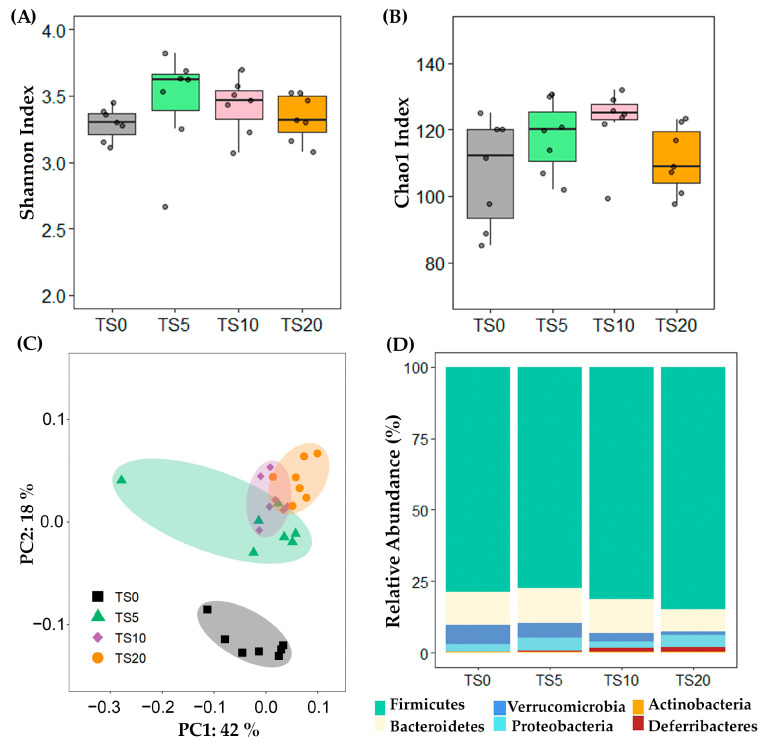
(**A**) Shannon and (**B**) Chao1 indices and (**C**) Principle Coordinate Analysis (PCoA) plot for the β-diversity and (**D**) bar chart for the relative abundance at the phylum level. For (**A**,**B**), dots show the value of each sample and data were analyzed using the non-parametric Kruskal–Wallis H test, followed by the Bonferroni correction. TS0, 0% turmeric starch; TS5, 5% turmeric starch; TS10, 10% turmeric starch; TS20, 20% turmeric starch; *n* = 7.

Alpha diversity at the feature level was determined using the Shannon and Chao1 indices. The Shannon (Figure 2A) and Chao1 indices (Figure 2B) were not significantly different among the diet groups. The PCoA plot revealed a clear differentiation in the microbial composition between the TS0 group and the other TS groups (Figure 2C). The PCoA plot implied that the TS diet had a significant impact on the gut microbial composition in the rats, indicating its influential role in shaping the microbial community.

Figure 2D shows the relative abundance at the phylum level. When compared with the TS0 group, the abundance of the phyla Firmicutes, Bacteroidetes, Verrucomicrobia, Proteobacteria, and Actinobacteria did not show significant differences among the groups. However, the phylum Deferribacteres was significantly higher (*p* < 0.05) in the TS10 and TS20 groups compared with the TS0 group.

**Figure 3:**
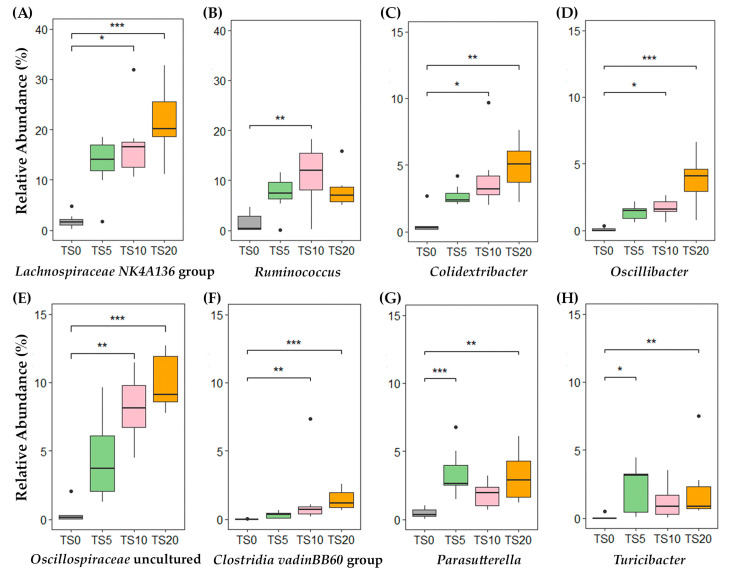
Relative abundances of selected microbial genera in cecal digesta of rats fed TS0, TS5, TS10, and TS20 diets for the 2 weeks of testing. Data were analyzed using the non-parametric Kruskal–Wallis H test, followed by the Bonferroni correction (* p < 0.05, ** p < 0.01, *** *p* < 0.001). TS0, 0% turmeric starch; TS5, 5% turmeric starch; TS10, 10% turmeric starch; TS20, 20% turmeric starch; *n* = 7.

The box and whisker plots obtained at the genus level (Figure 3) further highlighted the distinct cluster formation between the TS-fed groups and the TS0 group. In comparison to the TS0 group, the relative abundances of the *Lachnospiraceae NK4A136* group, *Clostridia vadinBB60* group, *Oscillibacter,* and *Oscillospiraceae* uncultured were significantly higher (*p* < 0.05) in the TS10 and TS20 groups, while showing a comparatively higher relative (*p* = 0.14, *p* = 0.32, *p* = 0.14, and *p* = 0.41, respectively) abundance in the TS5 group. Genera *Turicibacter* and *Parasutterella* showed significantly higher (*p* < 0.05) relative abundances in the TS5 and TS20 groups, with a relatively higher (*p* = 0.07 and *p* = 0.26, respectively) abundance in the TS10 group compared with the TS0 group. In addition, the relative abundance of the genus *Ruminococcus* was higher in the TS-fed groups, with a significantly higher abundance (*p* < 0.05) observed in the TS10 group than in the TS0 group. Moreover, the genus *Mucispirillum,* which belongs to phylum Deferribacteres, exhibited a significantly higher (*p* < 0.01) relative abundance (TS0 = 0.03%, TS5 = 0.22%, TS10 = 0.99%, TS20 = 1.42%) in the TS10 and TS20 groups compared with the TS0 group.

### 3.5. Intestinal Alkaline Phosphatase Activity

Remarkably, intestinal ALP levels vary along the longitudinal axis of the intestine. In this study, the duodenum showed significantly higher (*p* < 0.05) ALP activity only in the TS5 group compared with the TS0 group. In the jejunum, ALP activity was significantly lower (*p* < 0.01) in the TS10 and TS20 groups compared with the TS0 group. Similarly, in the ileum mucosa, ALP activity was significantly lower (*p* < 0.05) in all the TS groups compared with the TS0 group. However, the ALP activity of the cecum and colon mucosa was not affected by TS (Table 5).

In summary, TS supplementation significantly influenced gut health parameters and microbial composition in rats, exhibiting dose-dependent effects. Key results included: a decrease in visceral fat mass; increased cecal mucin content; increased cecal digesta mass and fecal dry matter; and elevated starch excretion. In addition, cecal acetate content increased significantly in a dose-dependent manner. The composition of the cecal microbiota shifted distinctly in the TS-fed groups, with an increased abundance of SCFA-producing bacteria such as the genera *Lachnospiraceae NK4A136* group and *Ruminococcus*.

## 4. Discussion

In recent years, dietary interventions have gained significant attention in influencing body composition and overall health. Among these interventions, diets supplemented with RS have shown significant results in reducing caloric intake and improving metabolic outputs. RS is a type of dietary fiber that resists digestion in the small intestine and is thus able to reach the colon, where it undertakes fermentation by gut microbiota [24]. This unique property of RS not only contributes to lower caloric density but also facilitates numerous health benefits, including improved gut health and reduced fat accumulation.

In this study, four TS-fed groups showed different effects on physiological parameters. Final body weight, weight gain, and feed intake were not significantly different from the TS0 group. This suggests that TS supplementation does not significantly affect overall growth or appetite. A notable finding, however, is the significant decrease in visceral fat mass in all the TS-fed groups, which can be attributed to the increasing levels of RS content in the diet (RS content/100 g diet: TS5, 3.09; TS10, 6.18; TS20, 12.4). These increases in RS likely reduced the energy density of the diet [25]. This energy dilution effect of the diet likely contributed to the reduced accumulation of visceral fat in rats [26], as evidenced by the positive correlation between caloric intake and visceral fat mass (*r* = 0.78, *p* < 0.01) [3]. Furthermore, starch excretion increased significantly and remarkably by 23% in the TS20 group in a dose-dependent manner. This massive increase in unabsorbed starch directly correlates with the increasing dietary levels of RS [27]. The TS20 group produced more than three times as much dry feces as the control group. This significant increase in fecal content is consistent with the known effects of RS, which can increase fecal bulk through fermentation by gut microbiota and undigested starch [24]. Similarly, this study showed a positive correlation between fecal dry weight and starch excretion (*r* = 0.96, *p* < 0.001). In addition, a significant increase could be observed in the cecal digesta mass in the TS-fed groups. It is likely that the substantial amount of RS contributed to the increase in cecal digesta volume and promoted microbial fermentation processes.

In addition to energy dilution, microbial fermentation of RS produces important SCFAs. These SCFAs have well-documented profound anti-obesity and anti-diabetic effects, regulating body weight and fat accumulation through the modulation of energy intake and expenditure [28]. Acetate, the most prevalent SCFA, may impact appetite regulation through a central homeostatic mechanism [29]. Although cecal acetate content increased significantly in a dose-dependent manner in this study, no significant correlation was found between feed intake and individual or total SCFA content. Interestingly, in this study, propionate and butyrate content were not significant among the diet groups, contrasting with a previous study [5] where rats fed with hydrothermally treated depigmented turmeric powder showed significantly higher propionate and butyrate content compared with the control group. Succinate is used to synthesize propionate via the succinate pathway [30]. In this study, lowered cecal succinate contents were observed in the TS-fed groups, which might indicate that succinate is further metabolized to propionate. Notably, cecal lactate content was significantly increased in the TS-fed groups compared with the TS0 group. Lactate is produced from rapidly fermentable indigestible saccharides [31] and can be converted into any major SCFA. The elevated lactate levels suggest that TS might have become a rapidly fermentable substrate in the gut environment.

The production of SCFAs not only influences the metabolic process but also has a significant effect on cecal pH. The pH of the cecum is critical for maintaining a microbial balance, and a slightly acidic environment is ideal for the fermentation process [32]. In this study, a significant dose-dependent decrease could be observed in the cecal pH of the TS-fed groups. This may be due to the increase in the production of organic acids in a dose-dependent manner that inversely impacted cecal pH levels [33]. Notably, cecal pH showed a negative correlation with total SCFA (*r* = −0.86, *p* < 0.001) as well as individual SCFA contents (acetate, *r* = −0.84, *p* < 0.001; propionate, *r* = −0.56, *p* < 0.01; butyrate, *r* = −0.47; *p* < 0.05).

In addition to SCFAs, mucin, IgA, and ammonia–nitrogen contents are also vital for a healthy intestinal environment [34]. Mucin forms a protective barrier in the intestine, preventing harmful substances from reaching the intestinal cells [35]. High RS content in the diet increases the bulk due to escape from digestion in the small intestine, physically stimulating the intestinal tract to enhance mucin secretion [18]. This can be attributed to the dose-dependent increase in cecal mucin content. Furthermore, SCFAs can stimulate mucin excretion by the goblet cells by influencing the expression of the mucin gene [36]. The findings of this study support this relationship, revealing positive correlations between cecal mucin content with the cecal acetate (*r* = 0.82; *p* < 0.01) and propionate (*r* = 0.69, *p* < 0.01) contents.

As the main component of mucosal immunity, IgA is an antibody that binds to pathogens and neutralizes them, thereby protecting intestinal epithelium invasion. The mutual interaction of mucin and IgA creates a cohesive immune barrier in the mucus layer, thereby preventing inflammation. For example, disruptions of mucin and IgA production could contribute to the development of gastrointestinal disorders [37]. Furthermore, IgA plays an important role in regulating the gut microbial composition of the gut, promoting bacterial symbiosis, and maintaining intestinal homeostasis [38]. A previous study reported [39] that acetate stimulates IgA secretion through GPR43-mediated (G protein-coupled receptor) mechanisms in dendritic cells, which then promote B cell differentiation and IgA production. This study also showed a positive correlation between cecal IgA contents and the cecal acetate (*r* = 0.78; *p* < 0.01).

Ammonia is produced in the intestinal lumen by two primary mechanisms—the degradation of amino acids and the hydrolysis of urea. Amino acids are derived from dietary protein or host cell turnover, and this process is facilitated by bacterial enzymes, while urea is broken down by the enzymatic action of ureases [40]. Increased cecal ammonia–nitrogen levels can have negative impacts on intestinal health and function, such as reducing the thickness of the cecal mucosa and muscle membrane, negatively altering the gut microbiota, and reducing growth performance [41]. This study showed slightly lower cecal ammonia–nitrogen contents in the TS-fed groups, which may be attributed to less amino acid fermentation by gut microbiota potentially contributing to lower ammonia–nitrogen levels [42].

The gut microbiota play a pivotal role in mediating many of the observed effects of dietary interventions, including the fermentation of RS and the production of SCFAs, while also influencing obesity or lean phenotypes, with reduced bacterial diversity being associated with obesity [43]. In this study, alpha diversity at the feature level did not differ significantly among the diet groups. This suggests that the overall diversity and distribution of the microbial community remained relatively consistent across the different doses of TS. Furthermore, this can also suggest that the TS diet did not negatively affect the gut microbial community in the rats. However, the PCoA plot revealed a distinct separation in the microbial composition between the TS0 and the other TS groups. Notably, the *Lachnospiraceae NK4A136* group, which belongs to the *Lachnospiraceae* family, emerged as the key player in the cecal microbiota of the TS-fed groups. The *Lachnospiraceae NK4A136* group is characterized by its anaerobic and spore-forming properties, enabling it to ferment complex polysaccharides into SCFAs efficiently [44]. In this study, the *Lachnospiraceae NK4A136* group showed a positive correlation with total SCFA (*r* = 0.62, *p* < 0.001) and acetate content (*r* = 0.58, *p* < 0.01). Further highlighting its potential to modulate the gut microbiota, a higher relative abundance of the *Lachnospiraceae NK4A136* group was reported to reduce the relative abundance of harmful microorganisms [45].

The *Ruminococcus* spp., a prominent member of the gut microbiota, plays a vital role in colonic fermentation by efficiently breaking down complex carbohydrates, including dietary fiber and plant polysaccharides that are typically resistant to digestion by the host [46]. In this study, the genus *Ruminococcus* exhibited a positive correlation with total SCFA (*r* = 0.46, *p* < 0.05) as well as acetate (*r* = 0.48, *p* < 0.01), propionate (*r* = 0.46, *p* < 0.05), and lactate (*r* = 0.53, *p* < 0.01) contents. *Colidextribacter, Oscillibacter,* and *Oscillospiraceae* uncultured belong to the family *Oscillospiraceae* which can ferment complex sugar into SCFAs [47]. Furthermore, a previous study has reported that the *Oscillibacter spp.* could enhance acetate production [48]; this study also showed that a positive correlation with cecal acetate content (*r* = 0.67, *p* < 0.001) could be observed. The genus *Colidextribacter* (*r* = 0.54, *p* < 0.01) and the genus *Oscillospiraceae* uncultured (*r* = 0.64, *p* < 0.001) also showed a positive correlation with cecal acetate content.

An elevated abundance of the genus *Parasutterella* has been associated with increased levels of acetate and propionate [49]. This study also exhibited a positive correlation with cecal acetate content (*r* = 0.42, *p* < 0.05). However, it did not show a significant correlation with propionate content. In addition, another SCFA-producing bacteria, the *Clostridia vadinBB60* group, showed a higher positive correlation with cecal acetate content (*r* = 0.72, *p* < 0.001) [50]. The genus *Turicibacter* showed a strong positive correlation with cecal lactate content (*r* = 0.72, *p* < 0.001); a previous study has identified lactate as the primary fermentative product [51], and this study similarly supports this finding. The genus *Mucispirillum* was reported to have both protective and potentially pathogenic effects in previous studies. It has been shown to protect against *Salmonella* infection [52]. However, *Mucispirillum* has also been associated with various inflammatory conditions, including inflammatory bowel disease, high-fat diet effects, and certain drug treatments [53]. These findings highlight the complex role of *Mucispirillum* in gut health, which may be context dependent. Consequently, the presence of these bacteria in the TS-fed groups might have played a pivotal role in the fermentation of RS and subsequent production of SCFAs, mainly acetate.

While this study observed significant changes in microbial composition and SCFA production, the effect of the TS diet on ALP activity in the intestine was observed. The ALP enzyme is widely distributed in various tissues, including the intestine, liver, kidney, and bone [54]. Intestinal ALP plays a critical role in maintaining intestinal health by multiple mechanisms. Intestinal ALP improves the intestinal barrier function by regulating the expression of tight junction proteins. Additionally, intestinal ALP dephosphorylates lipopolysaccharides of gram-negative bacteria [55]. Together, these actions contribute to the maintenance of a healthy gut microbiome and intestinal integrity [56]. A previous study reported that fermentable non-digestible carbohydrates increased colonic ALP activity [22]. However, the effects of specific compounds on ALP activity may vary. For instance, this study did not observe a positive effect of TS on colonic ALP activity, and changes in small intestine ALP activity are currently unclear. These results suggest that the precise mechanisms by which dietary factors influence ALP activity need to be further investigated.

In vivo studies using rats as models for colonic fermentation are ubiquitous due to the technical limitations and ethical concerns when involving human subjects. While it is important to acknowledge the differences between rat and human microbiomes [57,58], rats are considered to possess the closest gut microbiota resemblance to humans over other laboratory animal models (including guinea pigs, minipigs, swine, dogs, and non-human primates) [59]. However, we emphasize the need for further studies to identify suitable doses when introducing TS as a functional food for humans.

## 5. Conclusions

In conclusion, this study demonstrates that TS consumption, especially high in RS, exerts anti-obesity effects by reducing visceral fat mass and promoting beneficial changes in gut microbiota composition. Increasing the dose of the TS in the diet resulted in lower caloric intake, increased fecal dry weight, and decreased visceral fat mass and body weight, indicating improved energy metabolism. The production of SCFAs, in particular, increased acetate production in a dose-dependent manner, was positively correlated with mucin and IgA levels, suggesting the involvement of SCFAs in maintaining gut health and immune function. The enrichment of beneficial bacteria such as the *Lachnospiraceae NK4A136* group, *Ruminococcus* spp., and *Oscillospiraceae* uncultured further supported the beneficial effects of the TS diet on gut microbiota and SCFA production. Furthermore, while TS20 showed the most significant effects, it also resulted in potentially excessive starch excretion. TS5 and TS10 demonstrated many of the same benefits as TS20. Based on the current data, TS5 or TS10 might represent a more balanced approach, providing significant health benefits while minimizing nutrient loss through starch excretion.

## Figures and Tables

**Table 1:** Composition of crude turmeric starch.

Component	g Per 100 g
Moisture	9.74
Protein	1.43
Lipid	0.25
Ash	1.27
Total starch	68.80
Resistant starch	61.84
Digestible starch	6.96
Phosphorus (ppm)	4972
Energy (kcal per 100 g)	233.57

**Table 2:** Composition of the experimental diets.

Ingredients (g Per kg Diet)	TS0	TS5	TS10	TS20
Casein	200	200	200	200
L-Cystine	3	3	3	3
Soybean oil	70	70	70	70
Mineral mix (AIN-93G-MX)	35	35	35	35
Vitamin mix (AIN-93VX)	10	10	10	10
Choline bitartrate	2.5	2.5	2.5	2.5
Sucrose	100	100	100	100
3-Butylhydroquinone	0.014	0.014	0.014	0.014
Cellulose	50	50	50	50
Alpha-cornstarch	129.5	129.5	129.5	129.5
Cornstarch	400	350	300	200
Turmeric starch	0	50	100	200
Energy (kcal per kg diet)	3804	3730	3657	3509

TS0, 0% turmeric starch; TS5, 5% turmeric starch; TS10, 10% turmeric starch; TS20, 20%, turmeric starch.

**Table 3:** Effect of TS diet on body weight, fat accumulation, and fecal outputs.

	TS0	TS5	TS10	TS20
Final body weight (g)	239.90 ± 5.34	228.71 ± 2.00	232.19 ± 3.63	232.83 ± 4.96
Body weight gain (g/2 wk)	58.76 ± 2.05	48.43 ± 1.91	51.90 ± 1.88	52.40 ± 6.61
Feed intake (g/2 wk)	202.26 ± 3.67	193.84 ± 2.96	199.36 ± 2.66	202.09 ± 2.98
Perirenal + epididymal fat (g)	7.82 ± 0.89	5.32 ± 0.25 ***	5.56 ± 0.23 ***	4.84 ± 0.24 ***
Dry feces (g/2 d)	2.08 ± 0.06	3.62 ± 0.23 ***^,c^	4.72 ± 0.14 ***^,b^	6.94 ± 0.35 ***^,a^
Starch excretion (%)	0.04 ± 0.00	4.28 ± 0.56 **^,c^	8.95 ± 0.23 ***^,b^	23.0 ± 1.40 ***^,a^

Data are expressed as mean ± SE (TS0, 0% turmeric starch; TS5, 5% turmeric starch; TS10, 10% turmeric starch; TS20, 20% turmeric starch, *n* = 7). The statistical analysis was performed by one-way ANOVA, followed by Dunnett’s post hoc test (** *p* < 0.01, *** *p* < 0.001) to compare all TS diets with TS0 and by Tukey’s test (^a–c^ *p* < 0.05) to compare between TS-fed groups.

**Table 4:** Cecal parameters.

	TS0	TS5	TS10	TS20
Organic acid (µmol/content)				
Acetate	257.57 ± 19.49	407.86 ± 60.43 *	451.07 ± 37.38 **	544.57 ± 27.06 ***
Propionate	35.72 ± 5.65	40.17 ± 4.97	41.53 ± 3.59	46.93 ± 4.05
*n*-Butyrate	21.27 ± 5.15	27.73 ± 4.39	28.48 ± 6.61	27.08 ± 5.33
Total-SCFA	314.56 ± 28.03	475.75 ± 66.68 *	521.07 ± 41.13 **	618.57 ± 31.72 ***
Succinate	25.00 ± 9.35	7.86 ± 2.07	4.67 ± 2.04 *	5.62 ± 1.84 *
Lactate	2.41 ± 1.63	11.78 ± 2.96	13.56 ± 3.67 *	12.90 ± 2.24 *
Cecal pH	7.77 ± 0.04	7.40 ± 0.09 *^,a^	7.25 ± 0.05 ***^,a,b^	7.12 ± 0.05 ***^,b^
Cecal tissue (g)	0.68 ± 0.01	0.93 ± 0.07 **	1.07 ± 0.07 ***	1.11 ± 0.04 ***
Cecal digesta (g)	2.41 ± 0.25	3.54 ± 0.39 *^,b^	3.70 ± 0.28 *^,a,b^	4.71 ± 0.22 ***^,a^

Data are expressed as mean ± SE (TS0, 0% turmeric starch; TS5, 5% turmeric starch; TS10, 10% turmeric starch; TS20, 20% turmeric starch, *n* = 7). The statistical analysis was performed by one-way ANOVA, followed by Dunnett’s post hoc test (* *p* < 0.05, ** *p* < 0.01, *** *p* < 0.001) to compare all TS diets with TS0 and by Tukey’s test (^a,b^ *p* < 0.05) to compare between TS-fed groups.

**Table 5:** Intestinal ALP activity (unit/mg protein).

	TS0	TS5	TS10	TS20
Duodenum	301.38 ± 55.53	1167.47 ± 194.93 *	831.09 ± 179.73	899.65 ± 279.13
Jejunum	155.36 ± 14.15	118.74 ± 11.24	75.32 ± 10.75 ***	100.09 ± 8.67 **
Ileum	126.91 ± 14.99	76.33 ± 8.05 *	73.06 ± 12.29 *	61.62 ± 12.80 **
Cecum	37.71 ± 4.43	39.14 ± 4.61	37.05 ± 4.80	47.99 ± 5.59
Colon	51.33 ± 8.43	55.94 ± 21.87	51.14 ± 9.03	11.89 ± 2.97

Data are expressed as mean ± SE (TS0, 0% turmeric starch; TS5, 5% turmeric starch; TS10, 10% turmeric starch; TS20, 20%, turmeric starch, *n* = 7). The statistical analysis was performed by one-way ANOVA, followed by Dunnett’s post hoc test (* *p* < 0.05, ** *p* < 0.01, *** *p* < 0.001) to compare all TS diets with TS0.

## Data Availability

Dataset available on request from the authors.

## Supplementary Materials

The following supporting information can be downloaded at: https://www.mdpi.com/article/10.3390/metabo14110572/s1, 16rRNA sequencing data.
